# 

**Published:** 1896-06-06

**Authors:** 


**otices of Births, Deaths, and Marriages** are inserted in this column at a charge of 2s. 6d. for an announcement not exceeding four lines.

**Notice to Advertisers and Subscribers.**

All Advertisements, Orders for Copies of The Hospital and Business Communications should be addressed to **THE MANAGER, (Not to The Editor),** The Hospital, 428, Strand, London, W.C.

The Cost of Subscription, post free, is as follows:—Per week, 2½d.; per quarter, 2s. 8d.; per half-year, 5s. 3d.; per annum, 10s. 6d. Foreign:—Per Annum, 15s. 2d.; payable, in all cases, in advance.

Ready Shortly.

**"Burdett's Hospitals and Charities, 1896."**

Seventh year of publication. Over 1,000 pp. Scarlet cloth, gilt lettered. Price 5s. A most complete guide to British, Colonial, Indian, and American Hospitals, Dispensaries, Nursing and Convalescent Institutions, and Asylums. A few complete sets of the preceding six issues of the "Annual" may still be obtained on application to the Publishers, The Scientific Press (Limited), 428, Strand, London, W.C.

### NOTICE TO CORRESPONDENTS.

All MS., Letters, Books for Review, and other matters intended for the Editor should be addressed The Editor, The Lodge, Porchester Square, London, W.

The Editor cannot undertake to return rejected MS., even when accompanied by stamped directed envelope.

**NOTICE.—Vol. XIX. of "The Hospital" (October, 1895 to March, 1896), in handsome cloth binding, is now ready and on sale at the Office of this Journal. Price 7s. 6d. Cases for binding the Numbers contained in this Volume, 1s. 6d. Index to Vol. XIX., 2½d., post free.**

**The Monthly Part for June is now ready, price 6d., by post, 8d.**

### Scraps and Gleanings.

The plans for the new Northern Hospital at Liverpool have been selected.

The Huddersfield Workhouse Hospital has been extended by the erection of new maternity wards containing 14 beds.

The new County Monaghan Infirmary has recently been opened by Earl Cadogan. It seems rather curious to find that the new infirmary is in reality a transformation of the old gaol premises.

The first pharmaceutical register of Western Australia contains the names of sixty-six persons, fifty-eight of whom are registered as being members of the Pharmaceutical Society of Western Australia.

The membership of the Leicester Provident Dispensary has increased by about 25 per cent. since 1890, and now numbers no less than 40,325. Over a quarter of a million prescriptions were dispensed during the past year.

Funds are being raised to enable the South Wales and Monmouthshire Infirmary, at Cardiff, to open a new ward for women, the space available at present being quite inadequate for the number of cases waiting admission.

The "James Ward" at the North Devon Infirmary is to be utilised in future as a ward for children, with accommodation for twelve patients. This alteration will cost about £200 and will no doubt greatly enhance the popularity of the institution.

The Mercy Hospital, Cork, expended during the year 1895 £1,543, and received an income of £1,456, leaving a deficit for the year of £87. Five hundred and twenty-six in-patients were treated during the same period, and 21,390 attendances of out-patients were recorded.

Thirty-nine patients were admitted in 1895 to the hospital in connection with the Glasgow Public Dispensary, and 4,004 consultations were given. The income amounted to £424, of which £95 was received from annual subscribers. The expenditure amounted to about £440.

Three hundred and ninety-seven patients were admitted to the Suffolk Convalescent Home, Felixstowe, during the period the home was open last year, viz., from April 15th to December 23rd. Of these patients 54 obtained a second order extending their stay to six weeks, and one with three orders remained nine weeks.

The expenditure of the Nottingham and Midland Eye Infirmary exceeded the income by £117, owing chiefly to the increased cost of the free beds and to the purchase of additional furniture and household requisites. Three thousand two hundred and ninety-six out and 167 in-patients were treated during the year 1895.

Lady Augusta Mostyn, the president for many years of the Sarah Nicol Memorial Cottage Hospital at Llandudno, has been presented with an address setting forth the excellent work she has done for the hospital, and for the advancement of Llandudno generally. In addition to this her portrait has been presented to the hospital.

The Liverpool Hospital Saturday Fund collected in 1871, the year of its establishment, £104. Since then the collections have steadily increased, until in 1895 they have reached £5,012. Unfortunately, however, the Hospital Sunday Fund, which is worked by the same committee, has not flourished; in fact, the collection in 1895 was some £2,600 less than it was in 1874.

The Governors of the Carmarthenshire Infirmary have adopted a rule which effectually prevents any but the most important alterations being made therein, and then only if those who see the necessity for the amendment are public-spirited enough to put their hands in their pockets. The regulation is that persons wishing to alter any rule shall bear all the expenses of extra printing, &c., involved by such alteration.

A discussion has recently taken place in the Dundee newspapers as to the alleged difficulty of patients in obtaining admission to the Royal Infirmary, it being asserted that practically only patients having subscribers' lines were admitted. It is now stated that of 2,369 patients admitted during the year 1894-5 1,003 had subscribers' lines. Of the remainder 711 were admitted free and 655 as urgent cases.

The governors of the Cumberland Infirmary have been busily engaged recently on certain alterations in their statutes. The rules have now been amended so as to provide for the election to vacancies in the medical staff by a committee of election; for the retirement of the physicians and surgeons either on their attaining 66 or 60 years of age respectively or after 25 years' service; and for the election of assistant physicians and assistant surgeons for periods of seven years.

The daily average number of beds occupied at the Horton Infirmary, Banbury, during the year ended March 25th, 1896, was 22; 263 in-patients (of whom 62 were children under 12 years of age), 377 out-patients, and 410 dental cases were treated during the same period. In addition 806 patients were treated at the Provident Dispensary connected with the hospital. The income of the hospital was £1,016 (including £160 from legacies), and of the dispensary was £135, whilst the expenditure was £833 and £135 respectively.

The governors of the Buckinghamshire General Infirmary have been much exercised over the recent election of the house-surgeon. The result of the election turned on the validity of the proxies given for the successful candidate, and a special meeting was held last month to consider the matter. After a great deal of discussion, in which it was held that voting by proxy was allowable, a statement prepared by the secretary was put in, which showed that, after deducting the votes of governors who had not at the time of the election paid their subscriptions, the successful candidate still had a majority. The matter was therefore dropped.

We are sorry to read the following in the report of the Guest Hospital, Dudley, for the year ended September 30th, 1895: "The street collections have become an important feature in the income of the hospital. From £33 19s. in 1893, they have increased this year to £99 14s. 1d., and as there are several large districts which have not yet been included in the scope of this work we may confidently expect to see a still further increase." We should have thought that the experience in London and other large towns would have shown the managers of the hospital that, instead of seeking to extend these collections, it would be far better to do away with them altogether. May not the steady decrease in annual subscriptions and donations in the past two years be indirectly the result of this increased energy in the matter of street collections?

### BOOKS RECEIVED.

SIR JOSEPH CAUSTON AND SONS.

"Mountain, Moor, and Loch." Illustrated by Pen and Pencil, on the Route of the West Highland Railway. Second Edition.

A. AND C. BLACK.

"The Dream-Charlotte: A Story of Echoes." By M. Betham-Edwards.

MACMILLAN AND CO. (LIMITED).

"Physics for Students of Medicine." By Alfred Daniell.

### The Student of Medicine.

### APPOINTMENTS.

J. Adams, M.D., appointed Medical Officer of the Workhouse of the Eastbourne Union. A. Archibald, M.B., C.M.Aberd., appointed Assistant Medical Officer to the Gordon Road Workhouse and the Infirmary of the Camberwell Parish. R. H. F. Bostock, L.R.C.P., L.R.C.S.Edin., appointed Medical Officer for the Northern District of the Malton Union. F. Buckell, M.R.C.S.Eng., L.R.C.P., appointed Medical Officer of Health to the Borough of Romsey. R. Crawfurd, M.A., M.B.Oxon., M.R.C.P., appointed Physician to Out-patients at the Victoria Hospital for Sick Children, Chelsea. R. B. Eskrigge, L.R.C.P. Edin., M.R.C.S., appointed Medical Officer of Health to the Royston Urban District Council. Dr. Findlay, appointed Medical Officer to the Aberdeen Dispensary. F. S. D. Hogg, M.R.C.S., L.R.C.P.Lond., appointed Medical Officer for the Bradwell District of the Maldon Union. A. E. R. Hughes, M.R.C.S.Eng., appointed Assistant Medical Officer to the Gordon Road Workhouse and the Infirmary of the Camberwell Parish. F. A. Juckes, M.B., C.M.Edin., appointed Medical Officer of the Workhouse of the Horsham Union. J. G. Kay, M.B., C.M.Edin., appointed Medical Officer for the Tintern District of the Chepstow Union.

### VACANCIES.

The following vacancies are announced this week, the amount of salary in each case being given after the name of the institution:—

### Resident Medical Officers.

Bedford General Infirmary and Fever Hospital.—House Surgeon; doubly qualified. Salary £100 per annum, with apartments, board, lodging, and washing. Appointment for one year, but eligible for re-election. Applications to the Secretary by June 5th.

Borough Hospital, Birkenhead.—Junior House Surgeon; doubly qualified. Salary £60 per annum, with board and lodging, but no wine, spirits, or beer. A further sum of from £20 to £25 per annum is usually obtained in fees. Applications to the Chairman of the Weekly Board by June 9th.

Brentford Union.—Medical Superintendent of Infirmary and Medical Officer of Workhouse and Schools. Salary £250 per annum, with furnished residence in the infirmary, rations, washing, &c., or £300 with furnished residence, washing, &c., but without rations. Must be not less than 28 nor more than 40 years of age, and doubly qualified. Also Assistant Medical Officer. Salary £100 per annum, with furnished apartments in the infirmary, rations, washing, &c.; unmarried; doubly qualified. Applications on forms provided to be sent to William Stephens, Clerk to the Guardians, Union Office, Isleworth, W., by June 16th.

City of London Hospital for Diseases of the Chest, Victoria Park, E.—House Physician. Appointment for six months from July 1st. Board and residence provided, and salary at the rate of £30 per annum. Applications to the Secretary by June 11th.

Bucks County Lunatic Asylum, Stone, near Aylesbury.—Assistant Medical Officer; unmarried. Salary £100 per annum, with board and furnished apartments in the Asylum. Applications to Wm. Crouch, Clerk to the Visiting Committee, County Hall, Aylesbury, by June 5th.

Cancer Hospital (Free), Fulham Road, S.W.—House Surgeon. Appointment for six months. Salary at the rate of £50 per annum, with board and residence. Applications to the Secretary by June 6th.

General Hospital, Barbadoes.—Junior Resident Surgeon. Appointment for three years. Salary £200 per annum, payable monthly, with unfurnished quarters. Passage paid. Applications to the Secretary, Barbadoes General Hospital, by July 8th.

Govan District Lunacy Board.—Assistant to the Medical Superintendent at the Asylum at Hawkhead, near Paisley. Salary £100, with board and apartments; unmarried, and not more than 30 years of age. Applications to Andrew Wallace, Clerk, 7, Carlton Place, Glasgow, by June 10th.

North-Eastern Hospital for Children, Hackney Road, Shoreditch, N.E.—House Physician. Appointment for six months, at the expiration of which he will be required, if eligible, to serve as House Surgeon for six months. Salary as House Physician at the rate of £60 per annum; as a House Surgeon (the senior post) at the rate of £80 per annum. Must be doubly qualified. Junior House Physician; doubly qualified. Appointment for six months. No salary, but board and lodging (including washing) provided. Applications to the Secretary at the City Office, 27, Clement's Lane, E.C., by June 8th.

Royal Berkshire Hospital, Reading.—Assistant Medical Officer. Board, lodging, and washing provided. Honorarium of 10 guineas will be awarded if the duties are performed satisfactorily. Appointment for six months from July 1st. Applications to the Secretary before June 5th.

Royal Eye Hospital, Southwark.—Surgeons and Assistant Surgeons and Clinical Assistants. Applications to the Secretary at the hospital by June 8th.

### Non-Resident Medical Officers.

Charing Cross Hospital.—Surgical Registrar. Salary £40 per annum. Applications to Arthur E. Reade, Secretary, by June 8th.

Hospital for Sick Children, Great Ormond Street, W.C.—House Surgeon to Out-patients, non-resident. Appointment for six months, but eligible for re-election for a second term of office. Salary 25 guineas. Applications to the Secretary by June 16th.

London Hospital, Whitechapel, E.—Medical Registrar. Salary £100 per annum. Assistant Physician; must be M.R.C.P.Lond. Applications to G. Q. Roberts, Secretary and House Governor, by June 11th.

Manchester Clinical Hospital for Women and Children, Park Place, Cheetham Hill Road.—Honorary Assistant Physician. The selected candidate will not be allowed to hold any other hospital appointments. Applications to Mr. Hubert Teague, Secretary, 38, Barton Arcade, Manchester, by June 6th.

Middlesex Hospital, W.—Assistant Surgeon. Applications to F. Clare Melhado, Secretary Superintendent, by June 8th.

North-Eastern Hospital for Children, Hackney Road, Shoreditch, N.E. Physician to Out-patients; must be F. or M.R.C.P.Lond. Applications to T. Glenton-Kerr, Secretary, at the Office, 27, Clement's Lane, E.C., by June 10th.

